# Psychometric properties of the Chronic Rhinosinusitis Patient-Reported Outcome (CRS-PRO) questionnaire in a Spanish population

**DOI:** 10.1186/s41687-026-01023-2

**Published:** 2026-02-18

**Authors:** María Alharilla Montilla-Ibáñez, Javier Modesto García-Fernández, Ángeles Díaz-Fernández, María del Carmen López-Ruiz, Raquel Fábrega-Cuadros, Rafael Lomas-Vega

**Affiliations:** 1 Department of Otolaryngology, Complejo Hospitalario de Jaén, Jaén, Spain; 2 Department of Health Sciences, University of Jaén, Jaén, Spain

**Keywords:** Chronic rhinosinusitis, CRS-PRO, Patient-reported outcome, Questionnaire, Psychometric validation, Spanish adaptation, Nasal polyps, Rhinosinusitis

## Abstract

**Objective:**

To evaluate the psychometric properties of the Spanish version of the Chronic Rhinosinusitis Patient-Reported Outcome (CRS-PRO) measure in patients with chronic rhinosinusitis (CRS), including those with and without nasal polyps.

**Methods:**

A cross-sectional validation study was conducted with 124 CRS patients recruited from a tertiary otolaryngology center. Internal consistency (Cronbach’s α), test–retest reliability (ICC), construct validity (exploratory factor analysis), concurrent validity (Pearson correlation with NPQ, NOSE-E, and SNOT-22), and discriminative capacity (ROC curve analysis) were assessed. A subsample of 35 patients completed the CRS-PRO twice to evaluate reproducibility.

**Results:**

Factor analysis confirmed a three-factor structure (rhinologic symptoms, psychosocial impact, and facial discomfort), explaining 69.4% of the variance. Internal consistency was excellent (α = 0.874), and test–retest reliability was very high (ICC = 0.986; SEM = 2; MDC95 = 3). All items demonstrated moderate to high item-total correlations. The CRS-PRO strongly correlated with NPQ (*r* = 0.848), NOSE-E (*r* = 0.849), and SNOT-22 (*r* = 0.803). ROC analysis yielded an AUC of 0.929, with an optimal cut-off point of > 22 points (sensitivity 86.67%, specificity 86.08%) to identify severe symptom burden.

**Conclusions:**

The Spanish CRS-PRO is a valid, reliable, and efficient instrument for assessing disease-specific quality of life in CRS. Its strong psychometric performance, brevity, and diagnostic accuracy support its use in routine clinical practice, research, and digital health applications. These findings establish the CRS-PRO as a core PROM for Spanish-speaking patients with CRS.

**Clinical trial registration:**

Not applicable.

## Introduction

Chronic rhinosinusitis (CRS) is a persistent inflammatory condition of the nasal and paranasal mucosa, clinically characterized by symptoms such as nasal obstruction, rhinorrhea, facial pressure or pain, and a significant reduction or loss of smell lasting at least 12 weeks, as defined by the European Position Paper on Rhinosinusitis and Nasal Polyps 2020 (EPOS 2020). CRS affects up to 10% of the population in Europe and the United States, leading to a marked reduction in quality of life and considerable direct and indirect healthcare costs [1].

CRS is commonly classified into two major phenotypes: CRS with nasal polyps (CRSwNP) and CRS without nasal polyps (CRSsNP), which differ not only in clinical expression but also in pathophysiological and immunological mechanism [1]. This distinction is critical, as CRSwNP is often associated with greater disease burden due to anosmia, higher recurrence rates, and the need for surgical intervention.

Patient-reported outcome measures (PROMs) have become essential tools in both clinical practice and research, providing valuable insight into the subjective burden of disease and response to treatment. The Sinonasal Outcome Test-22 (SNOT-22) remains the most widely used PROM for CRS, with solid validation data and broad international use [2]. The Nasal Obstruction Symptom Evaluation (NOSE) scale [3] and the more recently developed Nasal Polyposis Questionnaire (NPQ) [4] have also contributed to the evaluation of sinonasal disease from the patient’s perspective. However, none of these tools were originally designed according to current FDA guidelines for PRO development, and most were developed prior to the establishment of modern consensus diagnostic criteria for CRS [1].

In response to these limitations, Ghadersohi et al. developed the Chronic Rhinosinusitis Patient-Reported Outcomes (CRS-PRO) instrument, a concise 12-item questionnaire specifically designed with input from patients diagnosed with CRS (both CRSwNP and CRSsNP) under current consensus criteria. The CRS-PRO demonstrated excellent internal consistency, test-retest reliability, and strong correlations with other health-related quality of life instruments, while reducing redundancy and cognitive burden compared to longer PROMs such as the SNOT-22 [5].

Following its original development, the CRS-PRO has undergone cross-cultural translation and validation into several languages, demonstrating its international applicability and robustness as a patient-reported outcome measure. Validated versions are currently available in French, Portuguese, Hebrew, and Arabic [6–9], all of which have reported favorable psychometric properties and supported the clinical utility of the instrument in diverse cultural and linguistic settings. These studies reinforce the relevance of the CRS-PRO as a standardized tool for assessing symptom burden and quality of life in patients with chronic rhinosinusitis across different populations.

Recently, a cross-cultural adaptation of the CRS-PRO into Spanish was conducted following international ISPOR guidelines. The study produced a semantically and culturally equivalent version of the instrument, suitable for use in Spanish-speaking populations. However, while the linguistic and conceptual adaptation has been completed, its psychometric properties (validity, reliability, and diagnostic performance) in this population have not yet been analyzed [10].

The aim of this study was to evaluate the psychometric properties of the Spanish version of the CRS-PRO, including internal consistency, construct validity, test-retest reliability, and its ability to discriminate between patients with different levels of symptom severity. This validation is essential for its implementation as a standard PROM in clinical and research settings for both CRSwNP and CRSsNP Spanish-speaking patients.

## Methods

### Design

To meet the objectives of this work, a cross-sectional validation study was designed. The protocol of this study was approved by the Research Ethics Committee of Andalusia (Spain) with code SICEIA-2024-001376. All the participants provided written informed consent to participate in this study, which was conducted in accordance with the Declaration of Helsinki, good clinical practices, and all applicable laws and regulations.

### Participants

To calculate the required sample size, the widely accepted criterion of including at least 10 participants per item of the questionnaire was applied, with a minimum of 80 subjects recommended for validation studies [11]. Considering that the CRS-PRO consists of 12 items, a minimum of 120 participants was required. A final sample of 124 patients was included to ensure adequate statistical power.

Participants were consecutively recruited from the Otorhinolaryngology Department of the Public University Hospital of Jaén (Spain), based on a confirmed diagnosis of chronic rhinosinusitis (CRS), with or without nasal polyps.

For the test–retest reliability analysis, a subsample of 35 patients completed the CRS-PRO questionnaire twice approximately one to two weeks apart, during which they remained clinically stable, with no changes in medical treatment and no reported acute exacerbations.

Inclusion criteria were patients aged 18 years or older who met the diagnostic criteria for chronic rhinosinusitis, with or without nasal polyps, in accordance with the definitions established by the European Position Paper on Rhinosinusitis and Nasal Polyps 2020 (EPOS 2020).

Exclusion criteria included: patients with neurological disorders, malignancies, acute infections, previous head and neck radiotherapy, or other major comorbidities that could confound the assessment of sinonasal symptoms.

### Measurements

The sociodemographic and clinical variables recorded included age (in years), gender (male, female, or nonbinary), weight (in kilograms), height (in meters, measured using a Detecto Model 2391 scale with integrated stadiometer), body mass index (BMI), and medical diagnosis (CRS with or without nasal polyps). These variables were collected and coded by a researcher independent of the diagnosis and participant selection processes.

The main instrument analyzed in this study was the CRS-PRO (Chronic Rhinosinusitis Patient-Reported Outcomes) questionnaire, a disease-specific tool designed to assess the impact of chronic rhinosinusitis on quality of life. It comprises 12 items grouped into three clinical domains:

- Physical symptoms (nasal obstruction, discharge, cough),
- Sensory function (sense of smell),
- Psychosocial impact (fatigue, emotional burden, sleep disturbance).

Each item is rated on a 5-point Likert scale ranging from 0 (“not at all”) to 4 (“as bad as it can be”), reflecting the severity of symptoms in the previous 7 days. The total score ranges from 0 to 48, with higher scores indicating a greater disease burden. The CRS-PRO has demonstrated strong responsiveness and validity in previous studies and is brief and easy to administer in clinical settings.

For concurrent validity analyses, the following validated instruments were also administered:

- the Sinonasal Outcome Test-22 (SNOT-22),
- the Nasal Obstruction Symptom Evaluation (NOSE-E),
- and the Nasal Polyposis Questionnaire (NPQ).

### Statistical analysis

Descriptive statistics were reported as means and standard deviations for continuous variables, and as frequencies and percentages for categorical variables. The normality of the data distribution was assessed using the Kolmogorov–Smirnov test.

Floor and ceiling effects were evaluated by counting the number of patients who obtained the lowest (0) and highest (48) possible scores on the CRS-PRO. These effects were considered present if more than 15% of participants achieved either extreme value [12].

The construct validity was analysed using principal component analysis (PCA) with Varimax rotation and Kaiser normalization. The adequacy of the data for extraction was confirmed using the determinant of the correlation matrix, Bartlett’s test of sphericity, and the Kaiser–Meyer–Olkin (KMO) measure. Although PCA is technically distinct from common factor analysis, it was selected to explore the underlying structure of the questionnaire, as commonly performed in psychometric validation studies, and terminology has been standardized throughout the manuscript for consistency.

Internal consistency was evaluated using Cronbach’s alpha coefficient, which assesses the extent to which items within a scale are correlated. Values below 0.70 were considered poor; values between 0.70 and 0.90, good; and values above 0.90, indicative of item redundancy [13, 14].

Test-retest reliability was analysed via the Shrouth and Fleiss Intraclass Correlation Coefficient (ICC) using an absolute agreement, two-way random effects model [15]. Reproducibility was considered poor when the ICC was < 0.40, moderate for values between 0.40 and 0.75, substantial for values between 0.75 and 0.90, and excellent when the value was > 0.90 [16]. The standard error of measurement (SEM) was calculated via the formula SEM = σ_base×√((1-CCI)), and the minimum detectable change (MDC) was obtained as the 95% confidence interval of the SEM (MDC95) with the formula 〖MDC〗_95 = Z × σ_base×√((1-ICC)), where “σ base” is the standard deviation of the pretest measures, the “ICC” is obtained from the test-retest reliability, and “Z” is the numerical value of the 95% confidence interval (CMD95), which is 1.96. The agreement between the two observations of each item was analyzed using the Kappa coefficient weighted by quadratic weights [17]. The agreement was considered null if Kappa < 0.00, insignificant if Kappa was between 0.00 and 0.20, discreet if Kappa was between 0.21 and 0.40, moderate if Kappa was between 0.41 and 0.60, substantial if kappa was between 0.61 and 0.80, and almost perfect if Kappa was between 0.81 and 1.00 [14].

Concurrent validity was assessed using Pearson’s correlation coefficient (r) by comparing CRS-PRO total scores with those of the NOSE-E and SNOT-22 questionnaires in the full cohort, and with the NPQ in patients with nasal polyps. According to Cohen’s guidelines, correlations were interpreted as strong (*r* > 0.50), moderate (0.30 ≤ *r* ≤ 0.50), or weak (*r* < 0.30) [18].

To assess the discriminative capacity of the CRS-PRO in identifying patients with severe symptom impact, a Receiver Operating Characteristic (ROC) curve analysis was performed. Severe symptom burden was defined using a SNOT-22 score ≥ 40 as the reference standard, in accordance with previously published thresholds indicating severe disease impact and EPOS 2020 recommendations [1]. The area under the curve (AUC) was interpreted using Swets’ criteria [19]: AUC > 0.90 = high discrimination; 0.70–0.90 = good; 0.50–0.70 = low; and ≤ 0.50 = no discrimination [20].

Optimal cut-off points for the CRS-PRO were calculated based on the ROC curve, using the SNOT-22 as the reference for severe symptom impact. At each threshold, sensitivity, specificity, positive predictive value (PPV), and negative predictive value (NPV) were calculated. Sensitivity was defined as the proportion of participants with severe symptoms correctly identified by the CRS-PRO, while specificity was the proportion of patients without severe symptoms correctly classified. PPV and NPV represent the probability that patients classified as positive or negative by the CRS-PRO actually had or did not have severe symptoms, respectively.

All statistical analyses were performed using IBM SPSS Statistics for Windows, version 27.0 (IBM Corp., Armonk, NY, USA) and MedCalc^®^ Statistical Software, version 22.021 (MedCalc Software Ltd., Ostend, Belgium; https://www.medcalc.org; 2024) by members of the research team with formal training in biostatistics, following established methodological guidelines for psychometric validation studies. A 95% confidence level was used, with p-values < 0.05 considered statistically significant.

## Results

**Fig. 1:**
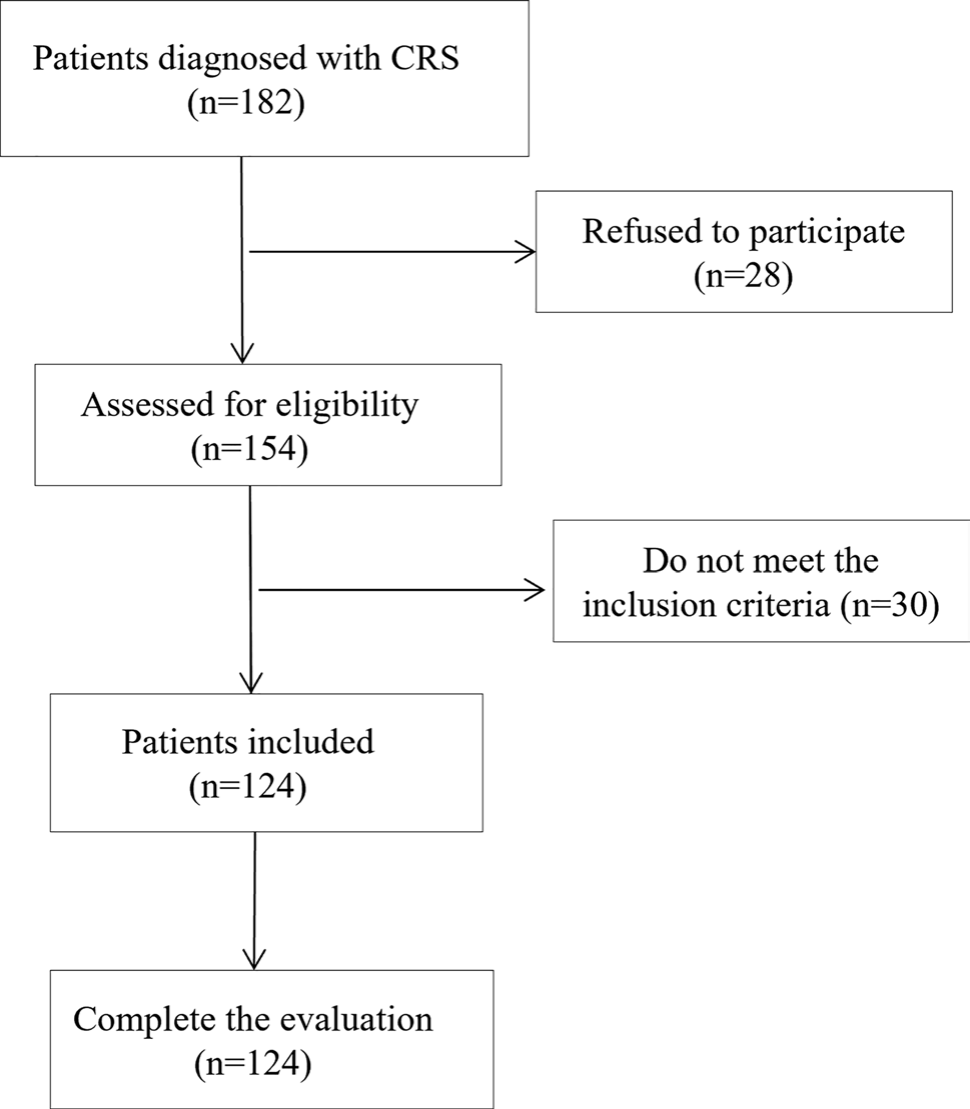
Flow diagram of patient recruitment

A total of 124 patients with chronic rhinosinusitis (CRS) agreed to participate in the study (Fig. 1). The mean age was 44.70 years (SD = 14.77), and the majority were male (56.5%). Table 1 shows the main sociodemographic and clinical characteristics of the sample. To assess reproducibility, a second administration of the questionnaire was performed in 35 patients, approximately one to two weeks after the first evaluation. No significant floor or ceiling effects were observed.

**Table 1:** Sample description

		COUNT	%	MEAN	SD
GENDER	Male	70	56.5%		
	Female	54	43.5%		
STUDY LEVEL	Primary education	58	46.8%		
	Secondary education	43	34.7%		
	Higher education	23	18.5%		
DIAGNOSTIC	CRSwNP	87	70.2%		
	CRSsNP	37	29.8%		
ALLERGIES	Yes	65	52.4%		
	No	59	47.6%		
ASTHMA	Yes	56	45.2%		
	No	68	54.8%		
AGE				44.70	14.77
WEIGHT				76.17	14.58
HEIGHT				168.85	10.41
BMI				26.70	4.39
SNOT 22				42.02	19.85
NOSE E				9.81	4.59
CRS-PRO				19.14	9.88

Exploratory factor analysis using principal component analysis (PCA) was conducted to examine the underlying structure of the questionnaire. A three-factor solution was extracted, accounting for 69.4% of the total variance (Table 2). The determinant of the correlation matrix was close to zero, suggesting that the matrix significantly deviates from an identity matrix and thus confirming the suitability of the data for factor analysis. The rotated component matrix (Table 3) revealed a clear factorial structure. The factors included items related to nasal and respiratory symptoms (items 1, 4–8), psychosocial and sleep impact (items 9–12), and facial pain/pressure (items 2–3), aligning with the original CRS-PRO subdomains.

**Table 2:** Explained variance by exploratory factorial analysis

Component	Initial Eigenvalues			Extraction Sums of Squared Loadings			Rotation Sums of Squared Loadings			
	Total	% of Variance	% Cumulative %	Total	% of Variance	% Cumulative %	Total	% of Variance	Cumulative %	
1	5.230	43.579	43.579	5.230	43.579	43.579	3.405	28.372	28.372	
2	1.820	15.163	58.742	1.820	15.163	58.742	3.104	25.863	54.235	
3	1.285	10.707	69.449	1.285	10.707	69.449	1.826	15.214	69.449	
4	0.771	6.429	75.877							
5	0.655	5.461	81.338							
6	0.558	4.646	85.985							
7	0.478	3.983	89.968							
8	0.339	2.828	92.796							
9	0.300	2.500	95.296							
10	0.243	2.021	97.317							
11	0.204	1.697	99.014							
12	0.118	0.986	100.000							

Extraction method: Principal Component Analysis

**Table 3:** Rotated component matrix with original english and Spanish CRS-PRO items

Item	Original English item	Spanish version	Component		
			1	2	3
1	I had difficulty breathing through my nose	He tenido dificultad para respirar por la nariz	0.592		
2	I felt pressure in my face	He tenido sensación de presión en la cara			0.849
3	My face hurt	He tenido dolor en la cara			0.867
4	I had to blow my nose	He tenido que sonarme la nariz		0.709	
5	I have been coughing	He estado tosiendo		0.721	
6	I had mucus in my throat	He tenido moco en la garganta		0.718	
7	I had mucus in my nose	He tenido moco en la nariz		0.801	
8	I had problems with my sense of smell	He tenido problemas con el sentido del olfato		0.747	
9	My symptoms kept me awake at night	Los síntomas me han tenido despierto por la noche	0.873		
10	I felt fatigued	Me he sentido fatigado	0.876		
11	I worried that my condition will get worse	Me preocupa que mi condición empeore	0.713		
12	I was frustrated by my condition	He estado frustrado por mi afección / condición	0.717		

Extraction method: Principal component analysis

Rotation method: Varimax normalization with Kaiser

a Rotation has converged in 5 iterations

Internal consistency analysis yielded a Cronbach’s alpha coefficient of 0.874, indicating excellent reliability and low item redundancy. The analysis of Cronbach’s alpha if individual items were removed (Table 4) showed that elimination of items 3 (“facial pain”) and 8 (“problems with smell”) slightly increased the alpha value, although not to a degree that would justify their exclusion.

**Table 4:** Item–total correlations and Cronbach’s alpha if item deleted

ITEM	Corrected item-total correlation	Squared multiple correlation	Cronbach’s alpha if element is removed
V_1	0.735	0.626	0.854
V_2	0.407	0.513	0.872
V_3	0.235	0.387	0.878
V_4	0.736	0.772	0.852
V_5	0.507	0.504	0.867
V_6	0.527	0.439	0.866
V_7	0.733	0.796	0.853
V_8	0.326	0.336	0.880
V_9	0.635	0.677	0.859
V_10	0.583	0.664	0.862
V_11	0.710	0.645	0.854
V_12	0.603	0.536	0.861

Squared multiple correlation reflects the proportion of variance in each item explained by the remaining items

Test-retest reliability analysis demonstrated excellent reproducibility, with an intraclass correlation coefficient (ICC) of 0.986 (95% CI: 0.972–0.993). The standard error of measurement (SEM) was 2 points and the minimum detectable change (MDC) was 3 points. Table 5 presents the test-retest reliability results for each item, all of which showed either “almost perfect” or “substantial” agreement, with ICCs ranging from 0.770 to 0.961.

Concurrent validity was supported by strong positive correlations between the CRS-PRO total score and the NOSE-E (*r* = 0.849, *p* < 0.001) and the SNOT-22 (*r* = 0.803, *p* < 0.001) in the overall cohort, as well as with the NPQ (*r* = 0.848, *p* < 0.001) in patients with nasal polyps, suggesting that the CRS-PRO is consistent with established measures of symptom severity and quality of life in CRS.

**Table 5:** Test-retest realiability

ITEM	Measure	Lower	Upper	Reliability
Ítem 1	0.961	0.920	1.000	Almost perfect
Ítem 2	0.833	0.759	0.907	Almost perfect
Ítem 3	0.888	0.795	0.980	Almost perfect
Ítem 4	0.818	0.710	0.926	Almost perfect
Ítem 5	0.770	0.636	0.903	Substantial
Ítem 6	0.926	0.879	0.974	Almost perfect
Ítem 7	0.910	0.854	0.965	Almost perfect
Item 8	0.916	0.855	0.978	Almost perfect
Ítem 9	0.941	0.905	0.977	Almost perfect
Ítem 10	0.899	0.843	0.955	Almost perfect
Ítem 11	0.917	0.867	0.968	Almost perfect
Ítem 12	0.957	0.925	0.988	Almost perfect
TOTAL	0.986	0.972	0.993	Excellent

**Fig. 2:**
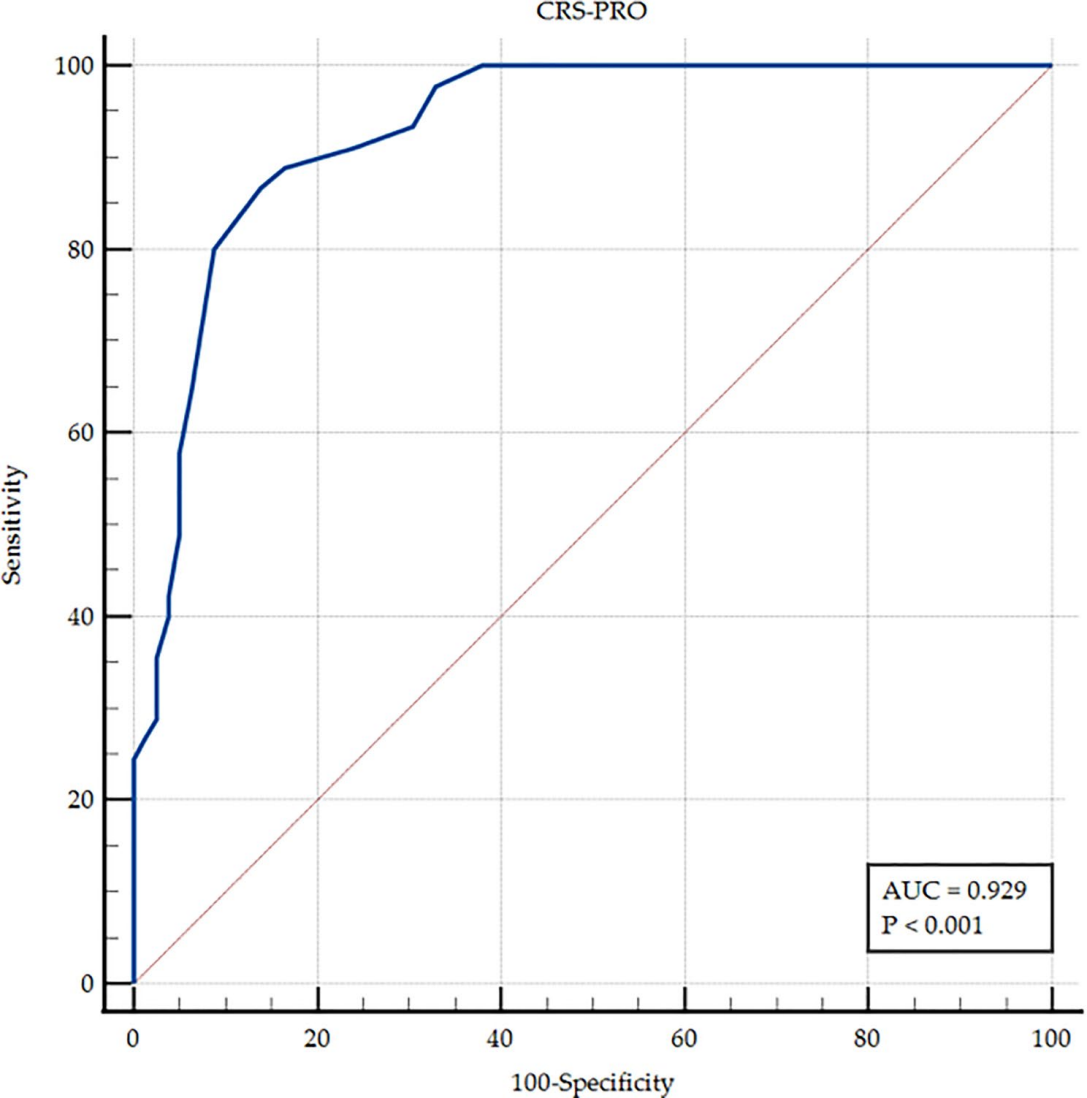
ROC curve

The ability of the CRS-PRO to discriminate between patients with and without severe symptom impact is illustrated in the ROC curve (Fig. 2). The total score achieved an area under the curve (AUC) of 0.929 (95% CI: 0.869–0.967; *p* < 0.001), indicating excellent discriminatory capacity. A threshold of > 22 points was identified as the optimal cut-off for detecting severe disease impact, yielding a sensitivity of 86.67% and specificity of 86.08%. Table 6 summarizes the predictive values, likelihood ratios, and confidence intervals derived from the ROC analysis.

**Table 6:** Predictive values

Criterion	Sensitivity	95% CI	Specificity	95% CI	+LR	95% CI	-LR	95% CI	+PV	95% CI	-PV	95% CI
> 22	86.67	73.2–94.9	86.08	76.5–92.8	6.22	3.55–10.90	0.15	0.073–0.33	78.0	66.9–86.1	91.9	84.3–96.0
> 23	80.00	65.4–90.4	91.14	82.6–96.4	9.03	4.39–18.59	0.22	0.12–0.40	83.7	71.4–91.4	88.9	81.6–93.5

**+**LR: positive likelihood ratio; –LR: negative likelihood ratio

## Discussion

This study presents the first formal psychometric validation of the Spanish version of the CRS-PRO, a 12-item patient-reported outcome measure designed specifically to assess the impact of chronic rhinosinusitis (CRS) on health-related quality of life. The results demonstrate that the Spanish CRS-PRO is a reliable, valid, and clinically useful tool for patients with both CRS with nasal polyps (CRSwNP) and without nasal polyps (CRSsNP).

Our exploratory factor analysis confirmed a three-factor structure—rhinologic symptoms, psychosocial impact, and facial discomfort—consistent with the original instrument developed by Ghadersohi et al. [5], and explaining 69.4% of total variance. This level of explained variance is considered strong for multidimensional PROMs.

The internal consistency observed in the Spanish version (Cronbach’s α = 0.874) is comparable to that reported for the original CRS-PRO (α ≈ 0.86) [5] as well as for other validated language versions, including the Portuguese [7] and French [6] adaptations, which have demonstrated similarly strong internal consistency and reproducibility and similar to other instruments such as the SNOT-22 or the Nasal Polyposis Questionnaire (NPQ) (2,4). Importantly, none of the items showed redundancy, as confirmed by corrected item-total correlations and alpha if item deleted analysis.

Item-total correlations supported the retention of all 12 items, as all showed moderate-to-high corrected correlations, with no evidence of redundancy.

Test–retest reliability was also excellent (ICC = 0.986), with individual items showing substantial to almost perfect agreement. The standard error of measurement (SEM = 2) and minimum detectable change (MDC95 = 3) were low, supporting the instrument’s precision for tracking symptom stability and change over time.

The CRS-PRO also showed strong concurrent validity, with robust correlations with the NPQ (*r* = 0.848), the NOSE-E (*r* = 0.849), and the SNOT-22 (*r* = 0.803). These results suggest that, although shorter, the CRS-PRO captures a similar construct of sinonasal disease burden as these more established instruments.

In terms of discriminative capacity, the Spanish CRS-PRO demonstrated excellent performance. The area under the ROC curve (AUC) was 0.929 for identifying patients with severe symptom impact, using the SNOT-22 as a reference. The validated cut-off point of > 22 showed high sensitivity (86.67%) and specificity (86.08%), reinforcing its clinical applicability. This cut-off could aid clinicians in identifying patients who may benefit from intensified therapy, including biological treatment or surgery, especially in alignment with EPOS 2020 criteria [1].

Unlike legacy instruments developed before current diagnostic consensus, the CRS-PRO was designed from the outset in alignment with modern criteria (EPOS 2020), incorporating patient input in its development process and focusing specifically on symptoms meaningful to patients’ quality of life [1, 5].

Compared to the SNOT-22, which includes 22 items covering both CRS-specific and non-specific symptoms (e.g., fatigue, sadness), the CRS-PRO is more focused, reducing noise from unrelated domains [2, 5]. The NPQ, although specific to CRSwNP, is longer (27 items) and includes broader psychosocial dimensions that may not be relevant for all patients (4). In contrast, the CRS-PRO was designed to be brief, clinically focused, and applicable to both phenotypes (CRSwNP and CRSsNP).

The brevity of the CRS-PRO (completion time < 2 min) increases its usability in routine care and helps reduce survey fatigue—a common limitation of longer PROMs such as the SNOT-22 [5, 10].

A recent predictive modeling study in CRSwNP patients showed that CRS-PRO scores could reliably estimate scores on other validated instruments, such as SNOT-22, NOSE, and NPQ, with excellent predictive accuracy (R² > 0.74) [21]. This finding supports the clinical interoperability of CRS-PRO with other widely used PROMs and may help facilitate harmonization in multicenter research or clinical registries.

Additionally, cross-cultural adaptations of the CRS-PRO into other languages, such as Portuguese [7] and French [6], have shown consistent psychometric performance. This reinforces the conceptual integrity and applicability of the instrument across diverse populations.

Although the CRS-PRO was not specifically designed to distinguish between CRS phenotypes, differences in scores between CRSwNP and CRSsNP patients suggest that it may reflect underlying disease burden variation. Future studies could explore its value in phenotypic stratification.

Given its psychometric performance, the CRS-PRO may prove particularly useful in evaluating longitudinal outcomes in clinical trials, stratifying patients for biological therapies, or facilitating remote monitoring via digital health platforms.

Limitations of the present study include the recruitment of patients from a single tertiary center, which may affect external generalizability, and the absence of responsiveness analysis, which is essential for confirming the instrument’s utility in monitoring clinical change. Future longitudinal studies should assess its sensitivity to medical and surgical interventions.

Nonetheless, this study adheres to internationally accepted guidelines for PROM validation, including ISPOR, COSMIN, and FDA frameworks [10, 22] and provides solid evidence supporting the use of the Spanish CRS-PRO in both clinical and research settings.

## Conclusions

The Spanish version of the CRS-PRO is a brief, reliable, and valid patient-reported outcome measure for evaluating the impact of chronic rhinosinusitis on quality of life. It exhibits excellent psychometric properties, including strong internal consistency, reproducibility, construct and concurrent validity, and high discriminative accuracy. The validated cut-off point, low respondent burden, and precise measurement error parameters support its use for individual symptom monitoring and clinical decision-making. Given its conceptual alignment with modern diagnostic standards and strong performance compared to legacy PROMs, the CRS-PRO may also facilitate outcome tracking in longitudinal studies and integration into digital health applications. These findings position the CRS-PRO as a cornerstone instrument for standardized symptom assessment in CRS across Spanish-speaking populations.

## Data Availability

The datasets generated and analysed during the current study are available from the corresponding author upon reasonable request.

## Abbreviations

AUC: Area under the curve
BMI: Body mass index
CI: Confidence interval
CRS: Chronic rhinosinusitis
CRSsNP: Chronic rhinosinusitis without nasal polyps
CRSwNP: Chronic rhinosinusitis with nasal polyps
CRS: PRO–Chronic Rhinosinusitis Patient–Reported Outcome questionnaire
EPOS: European Position Paper on Rhinosinusitis and Nasal Polyps
ICC: Intraclass correlation coefficient
KMO: Kaiser–Meyer–Olkin measure of sampling adequacy
MDC: Minimum detectable change
MDC95: Minimum detectable change at 95% confidence
NPQ: Nasal Polyposis Questionnaire
NPV: Negative predictive value
PCA: Principal component analysis
PPV: Positive predictive value
ROC: Receiver operating characteristic
SD: Standard deviation
SEM: Standard error of measurement
SNOT: 22–Sinonasal Outcome Test–22
SPSS: Statistical Package for the Social Sciences
Z: Z–score corresponding to 95% confidence (Z = 1.96)
